# Tuberculosis epidemiology and programmatic progress in the WHO Western Pacific Region, 2010–2024: an analysis of surveillance data

**DOI:** 10.1186/s41182-026-00971-1

**Published:** 2026-08-11

**Authors:** Fukushi Morishita, Kyung Hyun Oh, Machiko Otani, Amanda Reyes Veliz, Roger Evans, Huong Thi Giang Tran, Rajendra-Prasad Yadav

**Affiliations:** World Health Organization Regional Office for the Western Pacific, Manila, Philippines

**Keywords:** Tuberculosis, Western Pacific Region, Epidemiology, Surveillance

## Abstract

**Background:**

Tuberculosis (TB) remains a major public health challenge in the WHO Western Pacific Region, which accounts for over one quarter of the global TB burden. This study provides an updated regional assessment of TB epidemiology and programmatic performance to inform priority actions.

**Methods:**

We conducted a retrospective descriptive analysis of estimated TB burden and country-reported surveillance and programme data from the WHO global TB database, examining trends in TB epidemiology and programme performance in the Western Pacific Region between 2010 and 2024 with emphasis on progress since 2015 in line with the End TB Strategy. Analyses were undertaken at regional and country levels across 37 countries and areas, covering TB burden, case notifications, diagnostics, drug-resistant TB, TB–HIV care, treatment outcomes, TB preventive treatment (TPT), and costs faced by TB-affected households.

**Results:**

In 2024, an estimated 2.9 million people developed TB in the Region, with 227,000 TB-related deaths. Since 2015, TB incidence has stagnated (1.6% increase) and mortality has declined only modestly (1.3%). Following COVID‑19‑related disruptions, TB case notifications rebounded to near pre-pandemic levels by 2022, reaching 2.2 million in 2024; however, treatment coverage remained suboptimal at 75%. TB cases were concentrated in a small number of countries, led by Indonesia, the Philippines, and China. TB epidemiology was heterogeneous: children accounted for > 20% of notifications in several Pacific countries, older adults ≥ 65 years for > 50% in low-incidence economies, and foreign-born individuals for a large share of cases in migrant-receiving countries. Diagnostic performance improved substantially, with use of WHO‑recommended rapid diagnostics increasing from 4.2% in 2015 to 70% in 2024, and bacteriological confirmation from 43 to 59%. Detection of multidrug- or rifampicin-resistant TB doubled over the past decade. TB–HIV co‑infection accounted for 2.2% of notified TB, with HIV status ascertained in 70%, and ART coverage remaining at 50–69%. Treatment success for new and relapse TB declined slightly to 86–87% in 2022–2023. TPT enrolment increased more than 14-fold since 2015, yet coverage of eligible contacts remained low at 7.4%.

**Conclusions:**

Despite notable programmatic achievements in TB diagnosis, treatment and prevention, overall progress toward ending TB in the Western Pacific Region remains slow and uneven. Accelerating progress will require country-tailored strategies that respond to diverse epidemiological contexts and risk-group needs, alongside urgent action to close persistent case-detection and notification gaps, strengthen equitable TB care for vulnerable populations, and expand access to short‑course TPT.

## Introduction

Tuberculosis (TB) remains a major public health problem globally. In 2024, an estimated 10.7 million people developed TB worldwide, with the WHO Western Pacific Region accounting for 27% of the global burden [1]. The international community has committed to ending the TB epidemic by 2030, through the WHO’s End TB Strategy and the United Nations High-Level Meeting on TB, which set ambitious targets to substantially reduce TB incidence and mortality and to eliminate catastrophic costs faced by TB-affected households [2, 3].

Progress towards these targets depends not only on improvements in individual programme components, but on sustained performance across the full TB care and prevention cascade, from timely case detection and accurate diagnosis to effective treatment, successful outcomes and the delivery of preventive interventions. Over the past decade, substantial advances have been made globally in TB diagnostics, treatment regimens and preventive tools; however, scale‑up of these interventions has not consistently translated into proportional declines in TB incidence and mortality [1, 4]. This experience has underscored the importance of coverage, implementation quality, integration within health systems, and the ability to identify and address gaps across the cascade.

Routine TB surveillance data remain the primary means for monitoring progress, identifying performance gaps and informing corrective action at national and regional levels [5]. In the Western Pacific Region, surveillance systems have evolved substantially over time, enabling more comprehensive tracking of epidemiological trends and programme performance, including indicators related to diagnosis, treatment outcomes, drug‑resistant TB (DR-TB), TB‑HIV care and TB preventive treatment (TPT).

Despite important programmatic gains in several areas, a 2024 narrative review highlighted that overall progress towards End TB targets in the Region remains insufficient to meet agreed milestones, reflecting persistent implementation, health system, and equity challenges, compounded by the disruptive effects of the COVID‑19 pandemic [6]. In this context, up‑to‑date and comprehensive assessments of TB epidemiology and programme performance are essential to inform priority settings and monitor progress towards regional and global targets.

This paper synthesises estimated TB burden and country-reported TB surveillance and programme data for the period 2010–2024 to describe region-specific epidemiological patterns and programme performance, highlighting progress and remaining challenges, and inform priorities for strengthening TB control and elimination efforts in the Western Pacific Region.

## Methods

### Study design and setting

This study is a retrospective descriptive analysis of estimated TB burden and country-reported TB surveillance and programme data, examining TB epidemiological trends and pre-specified set of programme indicators in the WHO Western Pacific Region over the period 2010–2024. The study period was selected because consistent TB burden estimates and country-reported surveillance data are available across most countries and areas of the Region, with particular emphasis placed on progress since 2015 in line with the End TB Strategy. Analyses were conducted at both regional and country levels to characterise overall trends and examine heterogeneity within the Region.

The Region comprises 38 countries and areas, with a population of about 2.2 billion, and diverse epidemiological, demographic, and health system contexts. Pitcairn Island was excluded from analyses as it does not submit TB reports, given its population of fewer than 50 people, resulting in data from 37 countries and areas being analysed. Country-level analyses on overall case notification trends further highlighted eight regional priority countries; Cambodia, China, Indonesia, Lao People’s Democratic Republic (Lao PDR), Mongolia, Papua New Guinea, the Philippines, and Viet Nam, which together accounted for approximately 97% of the estimated regional TB incidence in the most recent reporting year.

### Data sources

Data were obtained from the WHO global TB database [1, 7], which compile annual country‑reported surveillance data. These data include estimates of TB incidence and mortality, case notifications, diagnostic testing, DR-TB, TB–HIV co‑infection, treatment outcomes, TPT, and costs faced by TB‑affected households. A full description of WHO’s data collection methods is available in the Global TB Report 2025 [1], and the methods used to estimate TB incidence and mortality are described in the accompanying online technical appendix [8]. In 2025, data for calendar year 2024 were reported to WHO by 31 countries and areas in the Region, accounting for 99.9% of the regional population. Indonesia, which officially transitioned from the WHO South-East Asia Region to the Western Pacific Region in May 2025, was included in this analysis using its complete set of country‑reported data for the period 2010–2024, consistent with the regional configuration adopted in the Global TB Report 2025. Accordingly, the regional aggregates presented in this study are not directly comparable with the regional figures published prior to 2025 [3, 6, 9].

### Data analysis

Data were analysed descriptively to derive programmatic indicators and assess temporal trends from 2010 to 2024, subject to data availability. Analyses were conducted at both regional and country levels and covered key TB epidemiological and programme indicators described above. Several WHO-recommended policies, guidelines and definitions evolved over the 15-year analytical window, including: introduction of rapid molecular diagnostics as the initial TB diagnostic test; revisions to TB treatment outcome definitions (2013 and 2021); introduction and scale-up of shorter all-oral MDR/RR-TB regimens; updated eligibility and regimen recommendations for TB preventive treatment (including shorter rifamycin-based regimens); and progressive expansion of the indicators collected through the WHO global TB data collection form. Indicators were therefore analysed under the definition in force in each reporting year. Indicators introduced after 2010 (e.g. coverage of WHO-recommended rapid diagnostic tests [WRD], treatment outcomes with the latest WHO definitions, TPT coverage) are reported from their first year of systematic availability (generally 2015). For indicators with incomplete country-level reporting in a given year, denominators were restricted to countries actually reporting in that year; no imputation was applied. No statistical modelling beyond WHO‑produced estimates was undertaken. All data management, analysis and visualization were performed using R software (version 4.5.1). All figures were generated de novo by the authors for this manuscript using the extracted WHO dataset. Figures were generated to illustrate trends over time and cross‑country variation in key indicators. In this paper, estimates are presented with 95% uncertainty intervals, referred to as “range” throughout the text.

#### Ethical considerations

This study used publicly available, aggregated secondary data, and no individual‑level data were accessed. Therefore, ethical review and informed consent were not required.

## Results

### Estimated TB burden

**Fig. 1:**
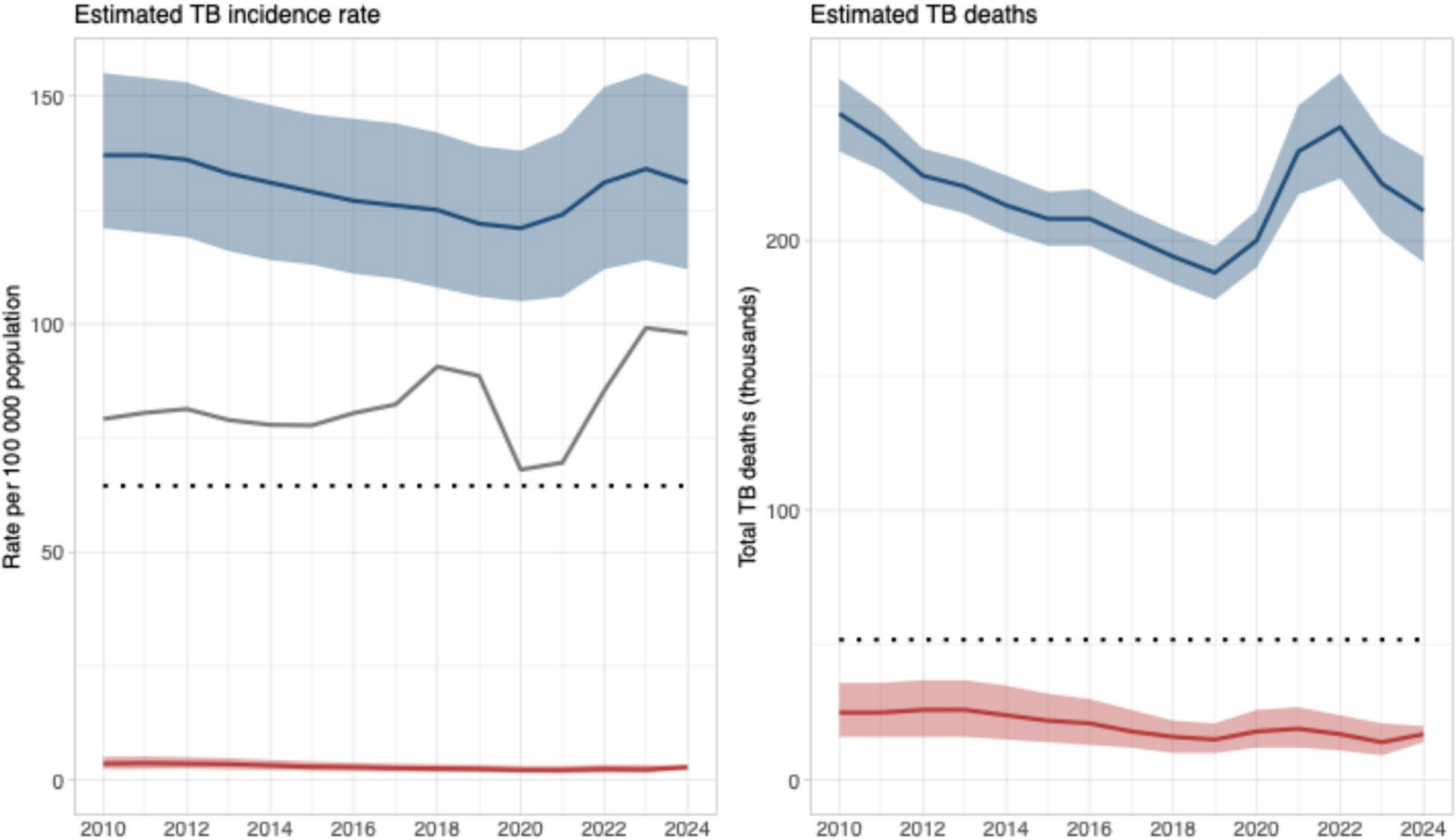
Estimated TB incidence rate and TB deaths in the Western Pacific Region, 2010–2024 Note: Estimated incidence and numbers of deaths are shown in dark blue and those among HIV-positive people in red. The horizontal dashed lines show the 2025 milestones of the End TB Strategy. Shaded areas represent uncertainty intervals. The grey solid lines show a case notification rate for new and relapse TB for comparison with estimated incidence rate

**Fig. 2:**
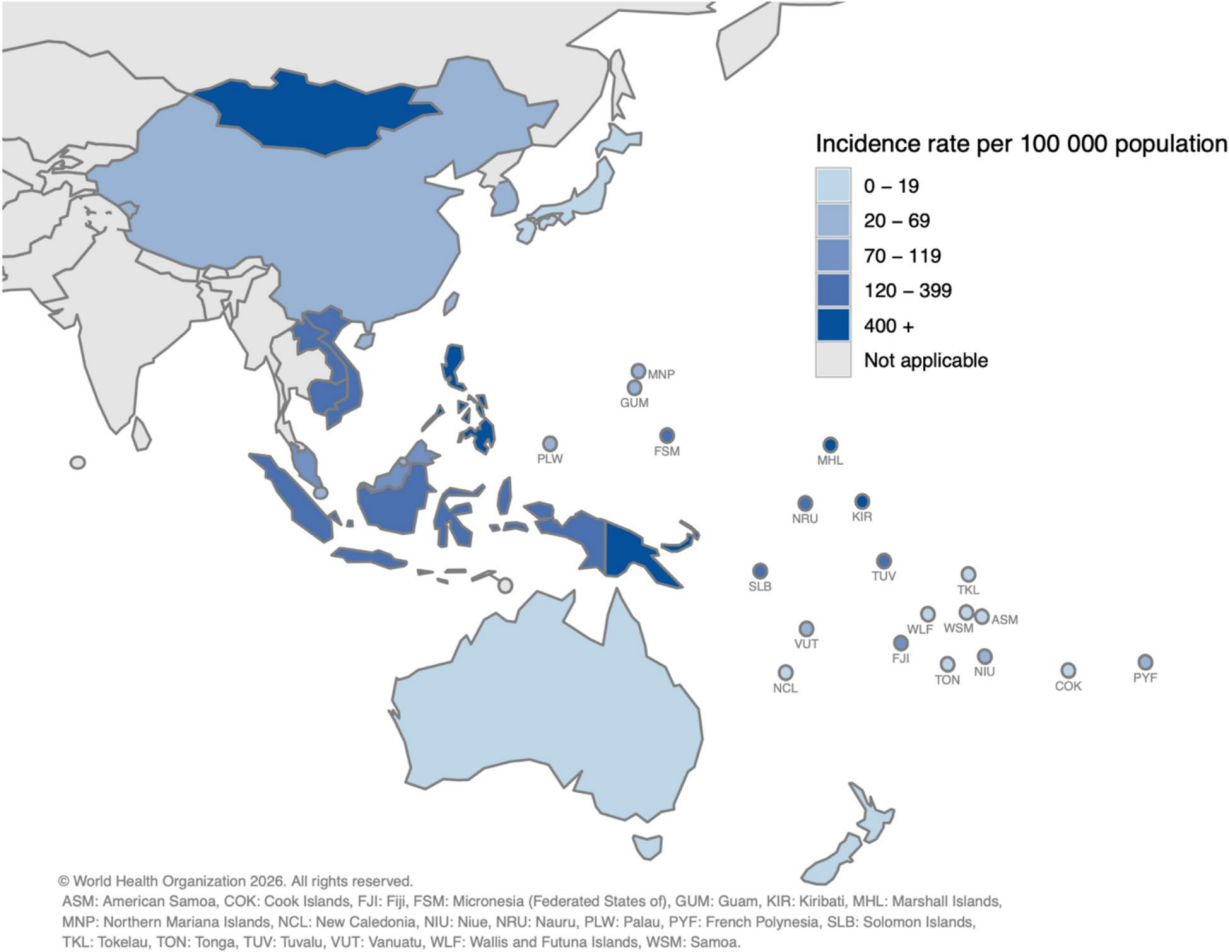
Map of estimated TB incidence rate in countries and areas of the Western Pacific Region, 2024. Disclaimer: The boundaries and names shown and the designations used on this map do not imply the expression of any opinion whatsoever on the part of the World Health Organization concerning the legal status of any country, territory, city or area or of its authorities, or concerning the delimitation of its frontiers or boundaries. Dotted and dashed lines on maps represent approximate border lines for which there may not yet be full agreement

**Fig. 3:**
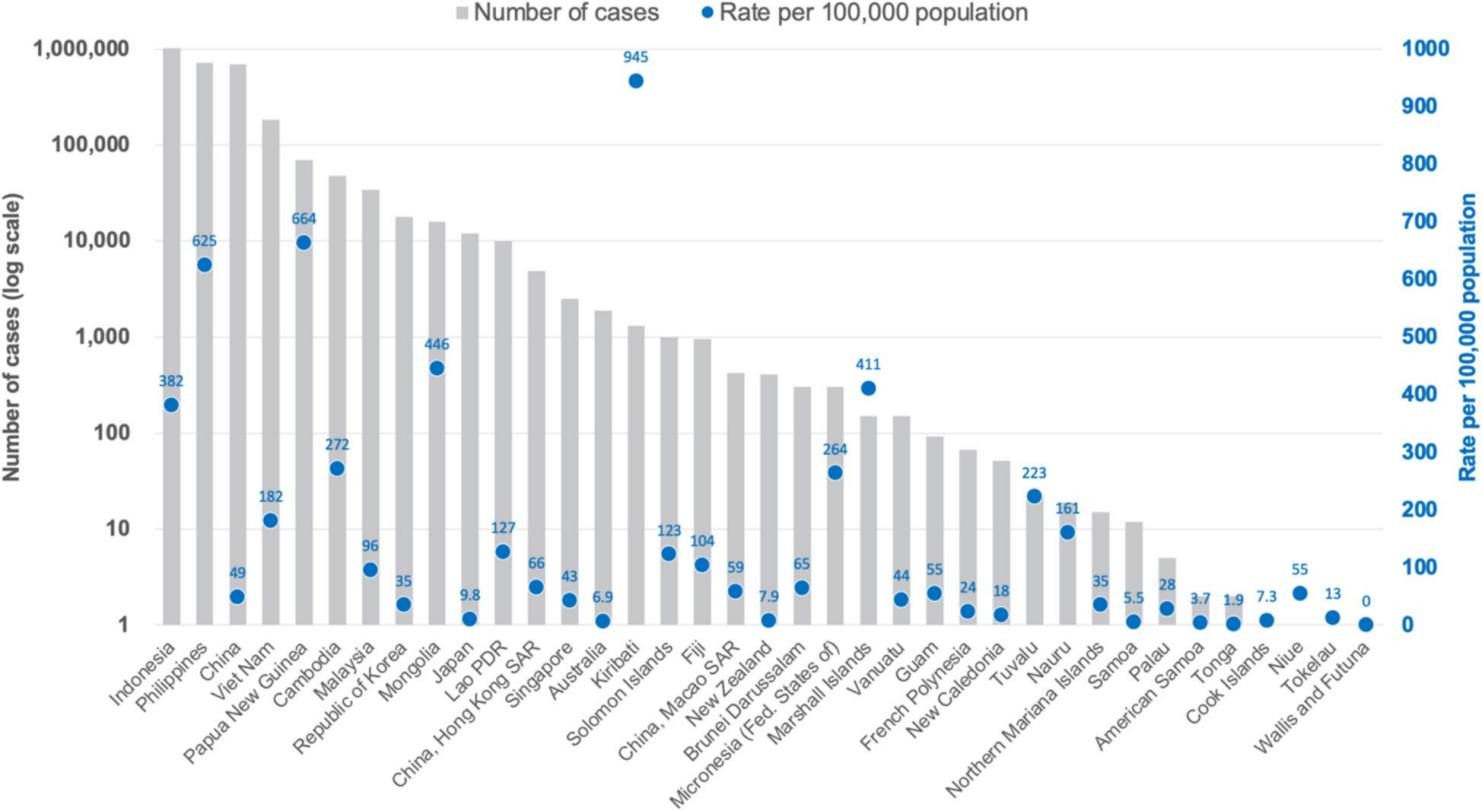
Estimated TB incidence in countries and areas of the Western Pacific Region, 2024. PDR: People’s Democratic Republic, SAR: Special Administrative Region

In 2024, the Region had an estimated 2.9 million people who developed TB (range, 2.5–3.4 million), corresponding to an incidence rate of 131 per 100,000 population (range, 112–152), and 227,000 TB-related deaths (range, 207,000–249,000). Since the launch of the End TB Strategy in 2015, the Region has seen a 1.6% increase in the TB incidence rate and only a modest 1.3% reduction in TB deaths by 2024 (Fig. 1). Substantial heterogeneity in TB incidence was observed across countries and areas of the Region (Fig. 2). Six countries, namely Cambodia, China, Indonesia, Papua New Guinea, Philippines and Viet Nam accounted for 96% of all incident TB cases in the Region (Fig. 3). Mongolia and Lao PDR also exhibited a high burden, with incidence rates exceeding 100 per 100,000 population and more than 5,000 estimated incident cases. Several Pacific island countries including Kiribati, the Marshall Islands, the Federated States of Micronesia, and Tuvalu had very high estimated TB incidence (> 200 per 100,000 population). In contrast, eight countries and areas had low estimated TB incidence (< 10 per 100,000 population), including American Samoa, Australia, Cook Islands, Japan, New Zealand, Samoa, Tonga and Wallis and Futuna.

### Case notifications

**Fig. 4:**
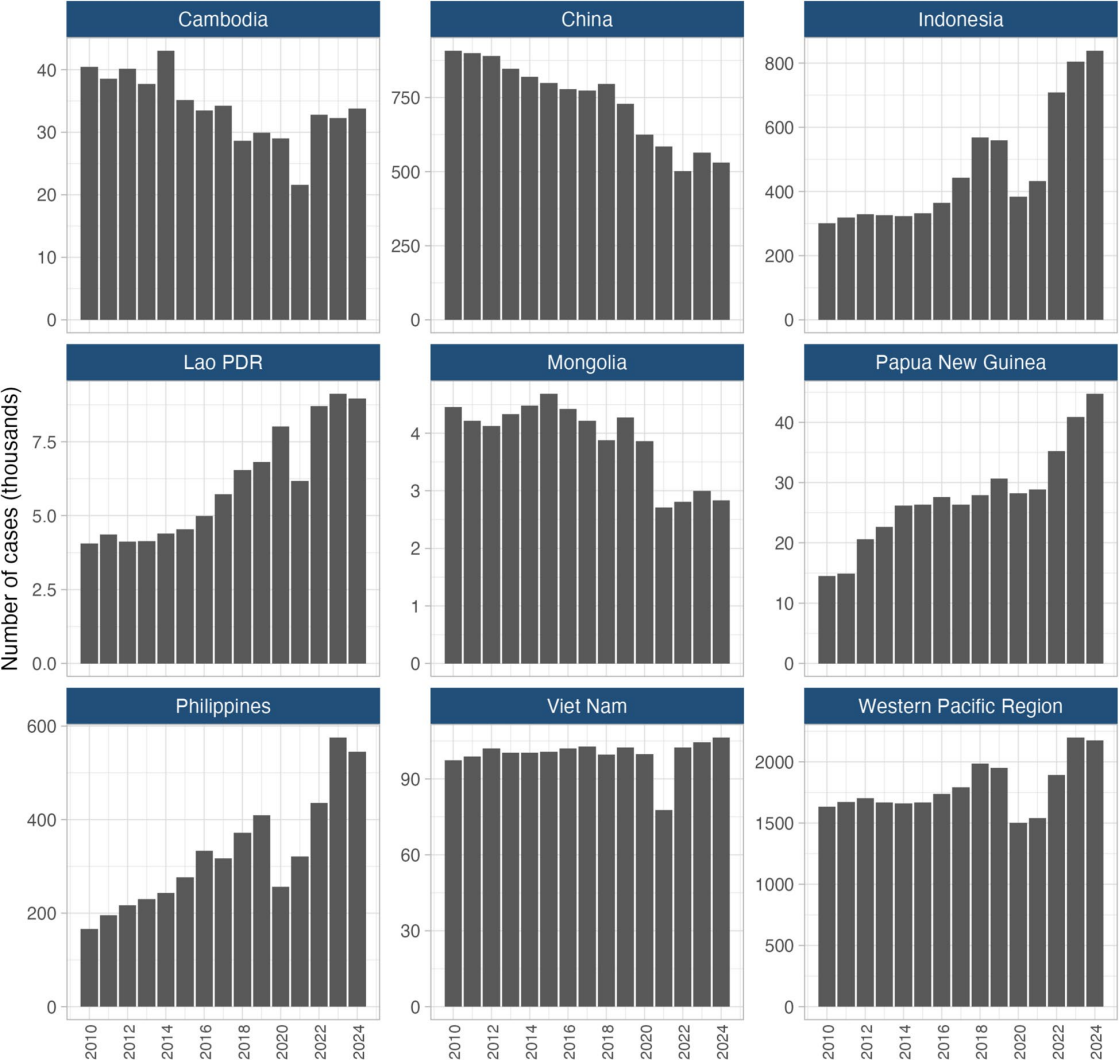
Number of notified TB cases (new and relapse) in the Western Pacific Region (bottom right) and in eight focus countries, 2010–2024. PDR: People’s Democratic Republic

Since 2010, the number of notified TB cases (new and relapse TB) in the Region remained relatively stable, ranging between 1.6 million and 1.7 million annually. This pattern persisted until 2015, after which case notification began to increase, driven largely by a substantial rise in notifications in Indonesia. In 2020, when the COVID-19 pandemic caused major disruptions to TB service delivery, case notifications declined by 23% compared to 2019 (from 1.95 million to 1.50 million) (Fig. 4). The decline in notifications continued through 2021. By 2022, regional TB notifications had largely recovered to near pre‑pandemic levels, reaching 1.89 million cases, followed by further increases in 2023 and 2024 to nearly 2.2 million cases. The rise is largely attributable to increased case notifications in Indonesia and Philippines (Fig. 4). Despite this recovery, TB treatment coverage remained suboptimal at 75% in 2024 (range, 64–88%), with an estimated one quarter of all people with TB remaining undetected or unreported. Table 1 presents a cross‑sectional snapshot of key TB notification, clinical and demographic characteristics at the country and area level for the most recent reporting year (2024), to provide context for the subsequent analyses. In 2024, Indonesia accounted for the highest number of notified TB cases in the Region (*n* = 839,018), followed by the Philippines (*n* = 544,948), China (*n* = 531,097), Viet Nam (*n* = 106,392), Papua New Guinea (*n* = 44,737) and Cambodia (*n* = 33,779) (Table 1). Together, these six countries accounted for 96.6% of all TB case notifications in the Region.

**Table 1:** TB case notifications, clinical and demographic characteristics, and TB-HIV indicators, by country and area in the Western Pacific Region, 2024

Country and area	New and relapse cases notified		Clinical characteristics		Demographic characteristics			TB-HIV	
	Number	Per 100,000 population	% extra-pulmonary TB	% MDR/RR-TB	% aged 0–14 years	% aged ≥ 65	% foreign born	% with known HIV status	% HIV + among TB patients (known status)
American Samoa	–	–	–	–	–	–	–	–	–
Australia	1,613	6.9	42	1.8	2	20	90	79	1.2
Brunei Darussalam	235	65	18	0.4	1.7	23	25	100	3.4
Cambodia	33,779	272	28	0.4	16	26	0	90	1.3
China	531,097	49	5.7	2.9	1.1	35	0	71	1.8
China, Hong Kong SAR	3,202	66	21	0.6	0.3	56	9.3	82	0.6
China, Macao SAR	276	59	12	3.6	0.4	45	15	98	1.1
Cook Islands	1	7.3	0	0	0	0	100	0	0
Fiji	567	104	23	0.9	12	9	1.4	100	28
French Polynesia	–	–	–	–	–	–	–	–	–
Guam	60	55	1.7	5.0	15	28	75	95	0
Indonesia	839,018	382	8	1.3	17	12	0	69	3
Japan	10,051	9.8	26	0	0.3	64	20	6.3	4.6
Kiribati	597	945	27	0.3	32	6.4	0	59	0
Lao People's Democratic Republic	8,965	127	5.3	0.4	0.7	25	0.1	89	4.2
Malaysia	26,183	96	15	0.7	5.8	17	16	100	4.6
Marshall Islands	92	411	30	1.1	19	8.7	3.3	99	0
Micronesia (Federated States of)	141	264	9.9	0.7	23	11	1.4	93	0.8
Mongolia	2,832	446	27	4.1	6.2	8.4	0.1	97	0.1
Nauru	–	–	–	–	–	–	–	–	–
New Caledonia	34	18	32	0	2.9	35	0	44	0
New Zealand	352	7.9	43	0	2.8	18	93	97	0.9
Niue	1	55	0	0	0	100	0	0	0
Northern Mariana Islands	10	35	0	0	20	10	50	80	0
Palau	5	28	0	0	0	0	80	80	0
Papua New Guinea	44,737	664	44	1.7	24	3.8	0	64	5.1
Philippines	544,948	625	1.8	1.4	8.5	18	0	67	0.8
Republic of Korea	16,798	35	22	2.7	0.2	59	6.4	0	0
Samoa	12	5.5	8.3	0	0	8.3	8.3	48	0
Singapore	2,238	43	13	1.0	0.1	32	54	98	0.8
Solomon Islands	453	123	29	0.2	6	7.5	0	0	0
Tokelau	–	–	–	–	–	–	–	–	–
Tonga	2	1.9	50	0	0	0	0	0	0
Tuvalu	–	–	–	–	–	–	–	–	–
Vanuatu	89	44	33	2.2	10	6.7	0	0	0
Viet Nam	106,392	182	16	3.4	1.2	22	0	80	2.8
Wallis and Futuna	–	–	–	–	–	–	–	–	–

MDR/RR-TB: multidrug- or rifampicin-resistant tuberculosis

The percentage of people with notified new and relapse TB who are children aged 0–14 years in the Region declined from 6–7% in 2017–2019 to 5.1–5.4% in 2020–2021 during the pandemic. It then rose to 8.9% in 2022 and 9.7% in 2024, with the highest number of childhood TB notifications recorded in 2023 (*n* = 215,170). This increase was largely attributable to Cambodia, Indonesia, Papua New Guinea, and the Philippines, which together recorded the greatest absolute increases in childhood TB notifications. Marked variation in the proportion of childhood TB was observed across countries and areas in 2023–2024, ranging from < 5% in more than 15 countries and areas to > 19% in five countries including the Marshall Islands (35%), Kiribati (31%), the Federates States of Micronesia (29%), Papua New Guinea (23%) and Cambodia (19%).

In 2024, nearly one in five (21%) people with notified new and relapse TB in the Region were aged 65 or older. The highest proportions were observed in Japan (64%), followed by the Republic of Korea (59%) and Hong Kong SAR (China) (56%) (Table 1), all of which are low or intermediate TB burden settings.

In 2024, TB among foreign-born individuals accounted for a small proportion of total case notifications at the regional level (0.5%). However, several countries with low and intermediate TB burdens reported much higher proportions of TB case notifications among foreign-born individuals during 2022–2024, including Australia (90%), New Zealand (90%) and Singapore (56%). In terms of absolute number, the highest numbers of notified TB cases among foreign-born individuals in 2024 were reported in Malaysia (*n* = 4285), Japan (*n* = 1980) and Australia (*n* = 1451).

### Diagnostic testing

In the region, the proportion of people newly diagnosed with pulmonary TB who were bacteriologically confirmed increased from 43% in 2015 to 59% in 2024. Over the same period, coverage of WRD for initial TB testing increased substantially, from 4.2% in 2015 to 70% in 2024. Cambodia, China and Viet Nam achieved marked increases in bacteriological confirmation rates during this period. In 2024, Lao PDR and Mongolia achieved WRD testing coverage for initial TB testing of 100% and 84%, respectively. However, despite expanded access to WRDs, Papua New Guinea and the Philippines reported bacteriological confirmation in less than 50% of people treated for pulmonary TB in 2024.

### Drug-resistant TB

**Fig. 5:**
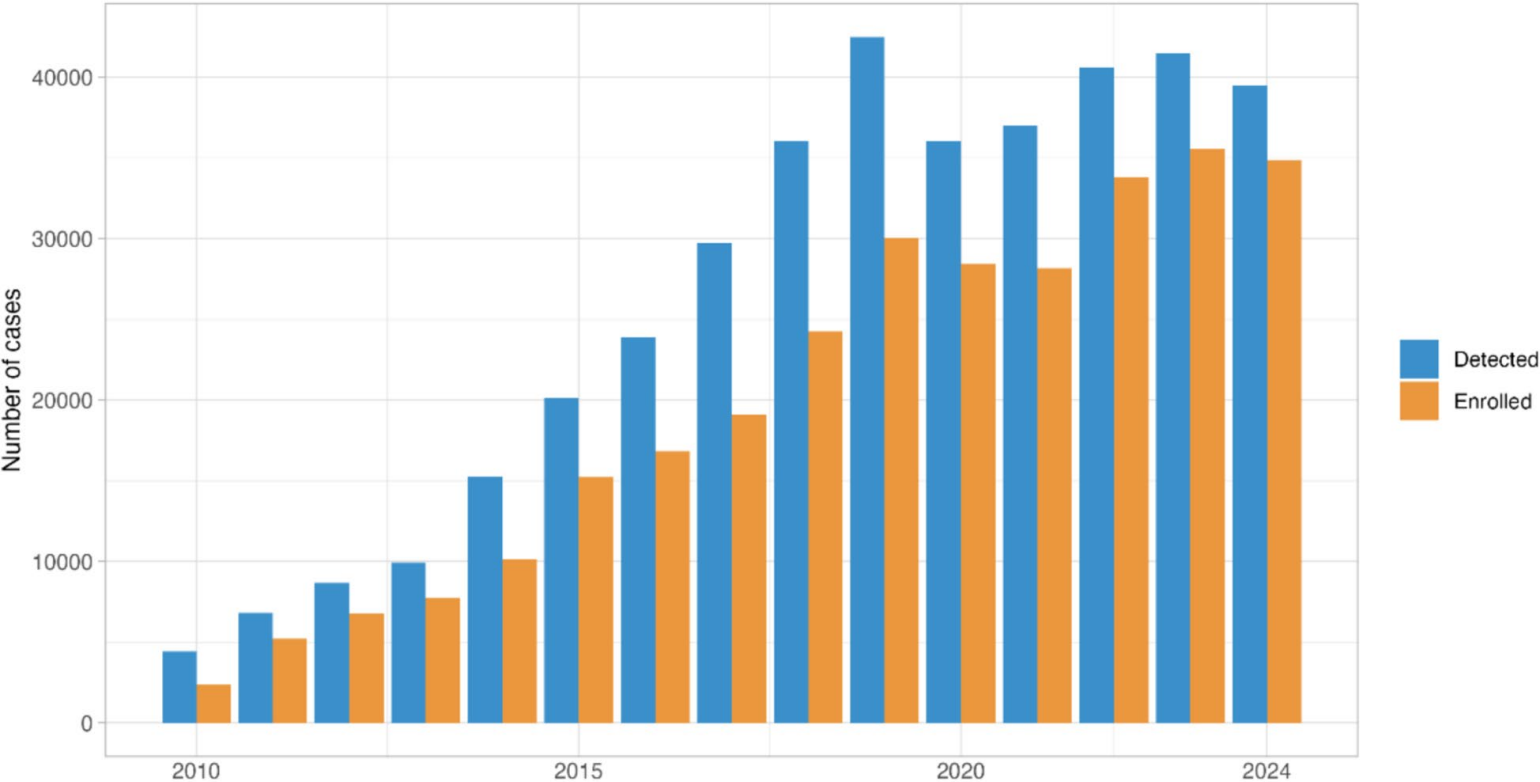
Number of MDR/RR-TB cases detected and enrolled on MDR-TB treatment in the Western Pacific Region, 2010–2024

The reported number of people diagnosed with multidrug- or rifampicin-resistant TB (MDR/RR-TB) in the Region nearly doubled from 20,129 in 2015 to 39,470 in 2024 (Fig. 5). In 2024, this represented approximately 40% (range 33–50%) of the estimated MDR/RR-TB incidence in the Region (98,000, range 80,000–120,000). In 2024, China reported the highest number of diagnosed MDR/RR-TB cases (*n* = 15,255), followed by Indonesia (*n* = 11,189), the Philippines (*n* = 7549), Viet Nam (*n* = 3658), and Papua New Guinea (*n* = 778). Together, these five countries account for 97% of all people diagnosed with MDR/RR-TB in the Region. The proportion of people diagnosed with MDR/RR-TB who were enrolled on appropriate treatment increased from 76% in 2015 to 88% in 2024. Notably, China recorded a marked improvement in treatment enrolment, rising from 46% in 2017 to 92% in 2024.

### TB-HIV co-infection and care

**Fig. 6:**
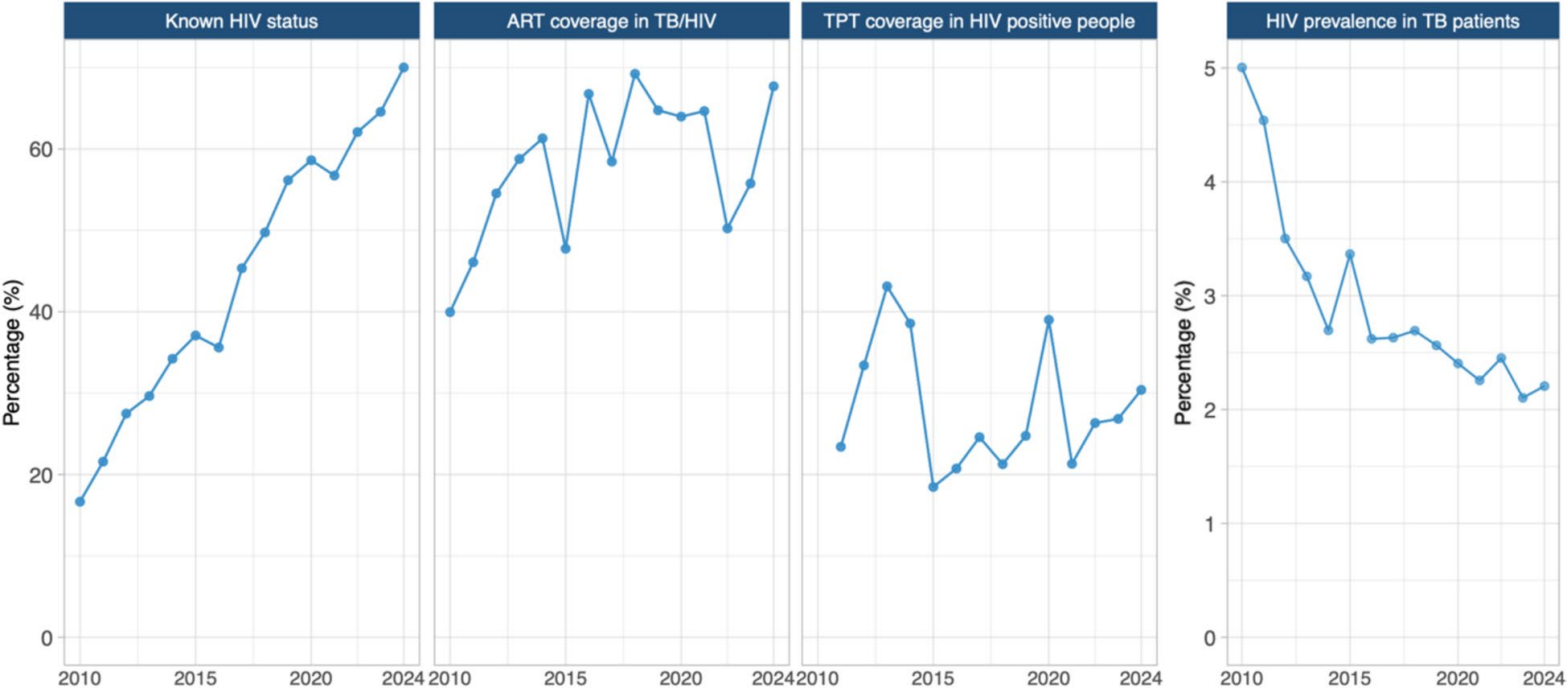
TB/HIV indicators in the Western Pacific Region, 2010–2024. TPT: Tuberculosis Preventive Therapy

In 2024, 33,344 people with new or relapse TB in the Region were living with HIV, representing 2.2% of TB cases with known HIV status. Key indicators for TB-HIV collaborative activities have improved over time (Fig. 6). The proportion of notified TB cases with known HIV status increased substantially, from 37% in 2015 to 70% in 2024. Among notified TB cases with known HIV status, HIV prevalence declined from 3.4% in 2015 to 2.2% in 2024. Coverage of antiretroviral therapy (ART) among TB/HIV co-infected patients remained below the global target of 90%, fluctuating between 50 to 69% since 2016. Similarly, coverage of TPT among people living with HIV (PLHIV) remained below 50% throughout the period. Nevertheless, the absolute number of PLHIV receiving TPT rose substantially from 8,393 in 2015 to 50,013 in 2024.

### Treatment outcomes

**Fig. 7:**
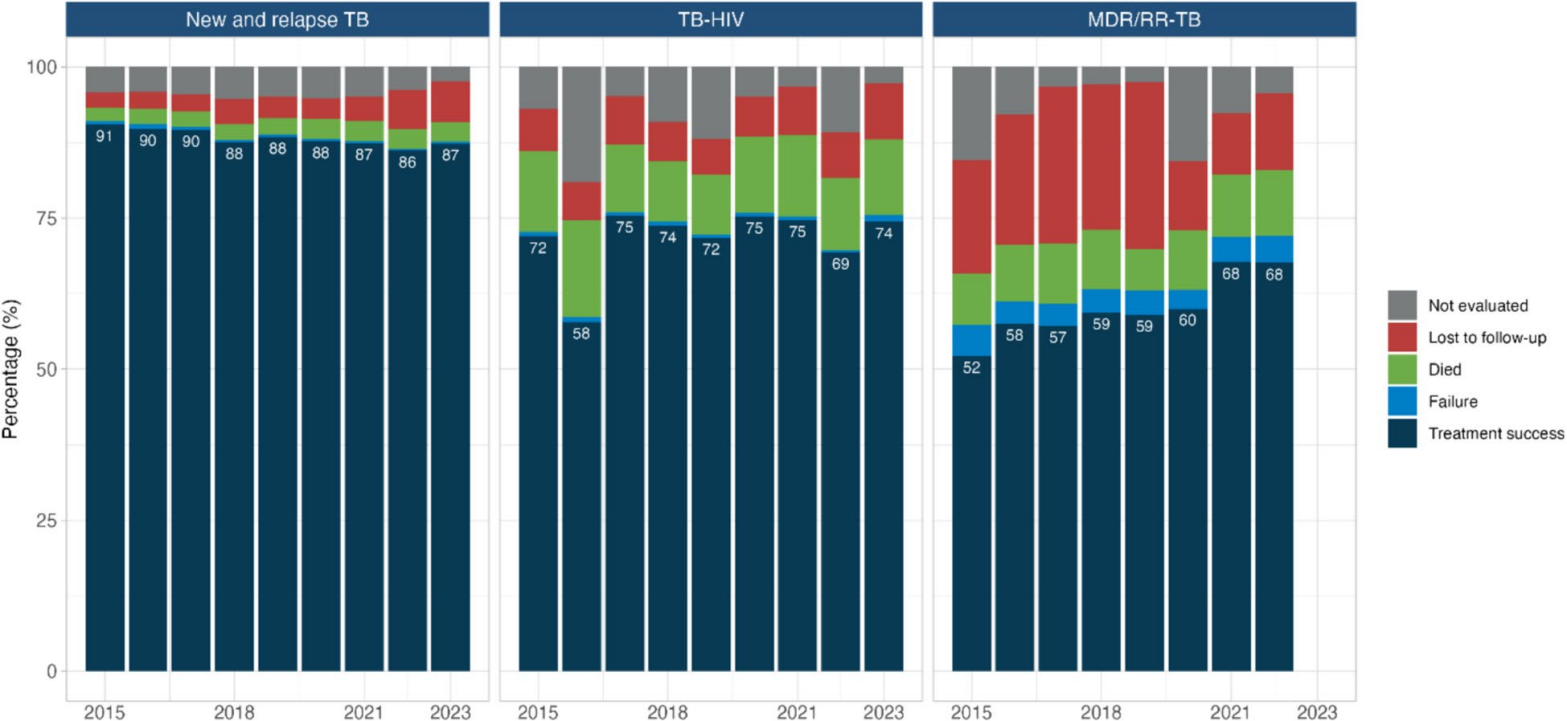
Treatment outcomes by type of TB in the Western Pacific Region, 2015–2023. MDR/RR-TB: multidrug- or rifampicin-resistant tuberculosis

The treatment success rate for people notified with new and relapse TB in the Region remained high at approximately 90% between 2015 and 2017, but declined to 86–87% in 2022–2023 (Fig. 7). Treatment success among people co-infected with TB and HIV remained consistently lower throughout the study period, with no notable improvement over time and a success rate of 74% in 2023. In contrast, the treatment success rate for people with MDR/RR-TB improved substantially, rising from 52% in 2015 to 68% in 2022.

**Fig. 8:**
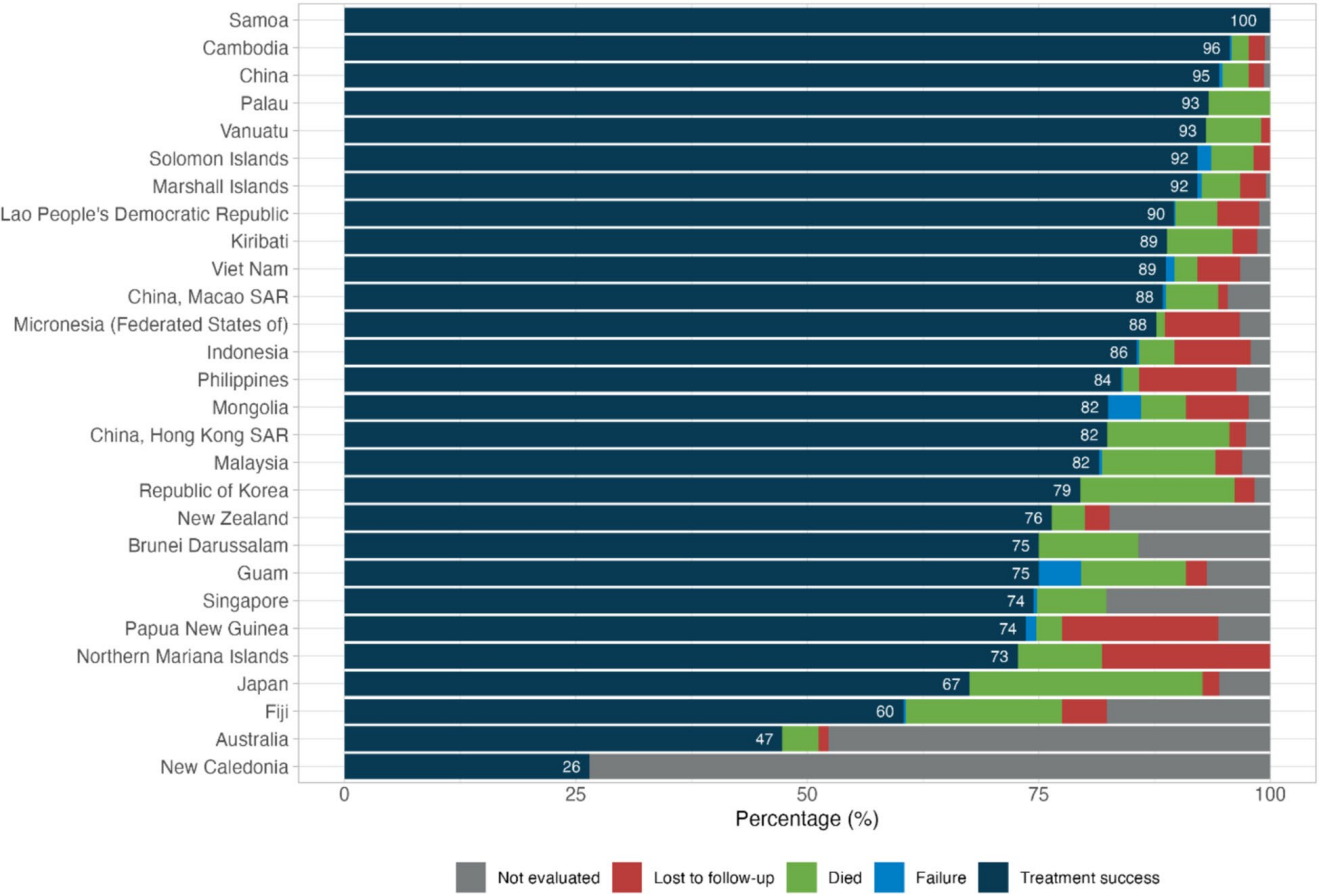
Treatment outcome for new and relapse TB by country and area in the Western Pacific Region, 2023. SAR: Special Administrative Region

For the 2023 patient cohort, 15 of 28 reporting countries and areas (54%) recorded treatment success rates below 85% (Fig. 8). Notably low success rates were observed in Fiji (60%), Japan (67%), and Papua New Guinea (74%). Countries where most notified TB cases are among foreign-born individuals also reported lower success rates, such as Australia (47%), Singapore (74%) and Brunei Darussalam (75%), with relatively high proportions of cases classified as “Not evaluated”.

### TB preventive treatment

**Fig. 9:**
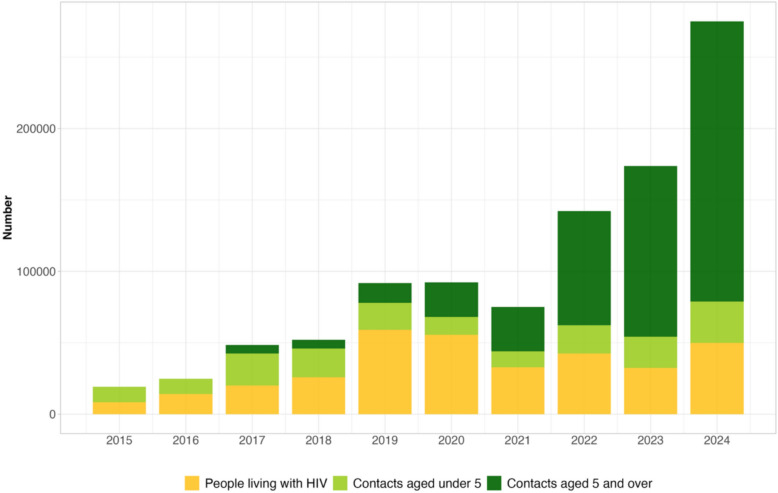
Number of people enrolled in TB preventive treatment in the Western Pacific Region, 2015–2024

The number of people enrolled on TPT in the Region increased more than 14-fold from 19,256 in 2015 to 274,998 in 2024 (Fig. 9). Much of the increase occurred in Cambodia, Indonesia, the Philippines, and Viet Nam, which together accounted for 86% of all TPT enrolments in the Region in 2024. Despite this progress, estimated TPT coverage among all household contacts in the Region remained critically low at 7.4% (range, 7.3–7.5%) in 2024. Coverage was similarly low among children under five years of age who were household contacts of bacteriologically confirmed pulmonary TB cases, with only 13% (range, 11–14%) received TPT across 16 countries with available data.

### Costs faced by TB-affected households

By the end of 2024, ten countries in the Region had conducted national surveys of costs faced by people treated for TB and their households, establishing a baseline for monitoring progress towards eliminating catastrophic costs due to TB. The surveys showed wide variation in the proportion of TB-affected households facing catastrophic costs, ranging from 34% (range, 31–38%) in Cambodia to 92% (range, 86–97%) in the Solomon Islands. Direct non-medical costs (such as transportation, accommodation and food) and indirect costs including income loss accounted for the largest shares of total costs reported by TB-affected households.

## Discussion

This study highlighted both notable progress and persistent challenges in TB control and elimination efforts in the Western Pacific Region. A major achievement in recent years has been the recovery of TB case notifications to pre-pandemic levels following the substantial disruptions caused by COVID-19, demonstrating the resilience of public health systems in the Region [10]. Progress in diagnostic performance has also been observed, including improved bacteriological confirmation rates alongside expanded use of WRDs for initial TB testing. These advances indicate broader access to, and more effective application of, improved microbiological diagnostic tools across many countries [11]. Despite the continued dual burden of TB and HIV in several settings, improvements have been observed in selected indicators, particularly HIV testing coverage among TB patients, alongside a decline in HIV prevalence among those tested. At the same time, treatment success rates for new and relapse TB have remained consistently high, demonstrating sustained programme performance for drug-susceptible TB. Encouraging progress has also been made in the management of MDR/RR-TB, with increased treatment enrolment and improved outcomes, likely reflecting the introduction and scale up of shorter MDR/RR-TB regimens (including BPaL/BPaLM) and expanded community-based treatment support in several countries [12]. In addition, TPT enrolment has increased substantially, largely driven by proactive implementation in high-burden countries, indicating greater emphasis on TB prevention.

Despite these achievements, challenges persist. An estimated one in four people with TB (25%) and three in five people with MDR/RR-TB (60%) in the Region remain undiagnosed, untreated or unreported, reflecting persistent gaps in case detection. Strengthening TB case finding efforts remains crucial especially in the high burden countries to ensure public health efforts to reduce TB burden are effective. Systematic screening targeting high-risk populations has been a core intervention within national strategic plans for TB in many countries for more than a decade [13, 14]. A range of active case finding (ACF) approaches has been implemented globally and within the Region at varying scale, contributing to detection of substantial number of people with TB [15–20]. Accumulated operational experiences and knowledge, together with advances in research and development, have led to optimized screening strategies including enhanced screening and diagnostic tools, and algorithms tailored to each specific risk group [14, 21]. However, growing evidence indicate that asymptomatic TB contributes substantially to ongoing transmission within communities [22]. This underscores the urgent need to consider population-wide systematic screening approaches, alongside the development of cost-effective and context-appropriate implementation models [23].

In addition, under-reporting of TB cases remains a major contributor to detection gaps, particularly in settings where healthcare providers, especially in the private sector, are not fully engaged in national surveillance systems [5, 24]. In the Region, the Philippines has a large and active private health sector involved in TB diagnosis and treatment and has historically accounted for a substantial proportion of unreported cases [25]. To address this challenge, the Department of Health introduced a mandatory notification policy and implemented a user-friendly national reporting system tailored to private providers, with simplified data entry focused on essential notification fields [5]. As a result, TB case reporting from the private sector increased markedly, with its contribution to total notifications rising from 9.7% in 2018 to 28.6% in 2022, although it declined slightly in 2023 (19%) and 2024 (20%) [7, 26]. In Indonesia, the second national TB inventory study conducted in 2023 demonstrated a substantial reduction in under‑reporting, from an estimated 41% in 2017 to 15.6% in 2023 (range: 13.4–18.2%) [27]. These improvements are attributed to strengthened surveillance systems, including implementation of a national digital reporting platform, legal mandates for universal TB notification, enhanced partnership with the national health insurance agency, and broader engagement with private-sector providers [27]. Building on these experiences, countries in the Region could further strengthen TB surveillance by systematically assessing under-reporting through record linkage across multiple data sources, thereby improving the completeness and quality of national TB surveillance systems [28].

TB epidemiology in the Region is highly heterogeneous, shaped by differences in population demographics, transmission dynamics, health system capacity, and population movement. In high-incidence settings such as Papua New Guinea and several Pacific island countries, a substantial proportion of notifications occur in children, indicating ongoing community transmission. While careful assessment is required to minimize the risk of overdiagnosis of childhood TB, ensuring accurate diagnosis, expanding decentralized childhood TB services, and building capacity to deliver optimal paediatric regimens remain essential priorities in these settings [29, 30]. In contrast, low‑incidence economies such as Japan, the Republic of Korea and Hong Kong SAR (China) report a higher proportion of cases among older adults. In settings with limited community transmissions, TB incidence among older adults is largely driven by reactivation of TB infection rather than recent transmission [31]. Given the rapid demographic ageing observed across much of the Region, older adults warrant specific consideration in TB response policies, including tailored diagnostic, treatment and care approaches [32].

Meanwhile, the number and/or proportion of TB in foreign-born individuals have increased in several countries receiving migrants from high burden countries, often occurring alongside a decline in TB incidence among nationals [33]. This pattern reflects changing population dynamics and presents distinct challenges for TB detection and care. Taken together, these diverse epidemiological profiles underscore the need for context-specific responses tailored to local transmission patterns, population structures, and health system capacities. Facilitating cross‑country learning, particularly by drawing on experiences from low‑incidence settings, may help inform adaptive strategies and strengthen TB responses across the Region.

The study identified a recent slight decline in treatment success among new and relapse TB cases at the regional level. This was largely driven by increased notifications from the private sector in the Philippines, which included a considerable proportion of TB patients classified as “Not evaluated” or “Lost to follow-up” [26]. Given the increasing role of the private sector in TB care across the Region, it is essential for national programmes to systematically engage and support private providers to deliver timely diagnosis, adhere to quality-assured treatment standards, and ensure reliable referral, reporting, patient follow‑up, and drug supply systems [34].

We also found that treatment outcomes among people co‑infected with TB and HIV remained consistently poor in the Region. People with TB/HIV co‑infection often present with more advanced disease and comorbidities, face higher risks of adverse drug reactions and drug-drug interactions, and require coordinated management across TB and HIV services [35]. In many regional priority countries, TB treatment for drug-susceptible TB is decentralized to peripheral health facilities, whereas ART services remain relatively centralized at the provincial/district or higher levels. This service configuration may contribute to delays in HIV diagnosis, incomplete linkage to ART, and interruptions in care, thereby compromising treatment outcomes. In addition, national household cost surveys indicated that social and financial barriers to care are greater among households affected by TB-HIV co-infection than among those affected by TB alone [36, 37]. Addressing these clinical, programmatic and socio-economic challenges will be essential to improve outcomes for this vulnerable population.

The study further showed that more than half of countries and areas in the Region reported suboptimal treatment success rates, highlighting persistent challenges in achieving favourable outcomes. In high- or intermediate-burden settings such as Papua New Guinea and Fiji, lower treatment success appears to reflect programme-level constraints, including limited community-based patient support and geographical barriers that hinder treatment supervision and follow‑up. In contrast, in countries with a higher proportion of cases among older adults, such as Japan where more than 50% of newly notified TB cases occur in people aged 75 or older [38], elevated mortality rates were observed. Improving treatment outcomes in this population requires a person-centred approach that integrates TB care with management of comorbidities, complemented by early monitoring for adverse-event and timely regimen adjustments to minimize drug-related toxicity [31]. Importantly, these countries have also developed substantial expertise in geriatric TB care, offering valuable insights for settings undergoing similar demographic transitions [32]. Conversely, countries with a higher proportion of foreign-born TB cases reported relatively high rates of lost to follow-up. Factors such as stigma, financial hardship, and visa-related concerns can delay diagnosis and disrupt treatment adherence in migrant populations [39]. Addressing these barriers requires culturally sensitive, linguistically appropriate services, and robust cross-border referral mechanisms to ensure continuity of care. While such population-specific interventions are critical, sustained efforts are also needed to deliver essential TB services through primary health care, maintain high standards of care in facilities outside national TB programmes, and strengthen community-based patient support. In this context, social protection measures, such as transport subsidies and nutritional assistance, can play an important role in improving treatment completion, particularly among vulnerable populations [40].

The number of people enrolled on TPT in the Region increased substantially. This increase was largely driven by greater TPT uptake among household contacts aged five years and older, coinciding with the expansion of TPT eligibility to include household contacts of all ages of people with bacteriologically confirmed pulmonary TB, as well as selected clinical risk groups [41]. Despite such notable scale-up of TPT, coverage among eligible contacts remains critically low across the Region, underscoring the need for sustained investment and targeted action to reduce TB infection among populations at highest risk. Increasing TPT uptake requires a multi-pronged approach, including strengthening systematic contact investigation, expanding access to TB infection testing, and improving the acceptability and delivery of TPT particularly for children and other high-risk groups [42]. Building on progress in countries such as Indonesia, the Philippines, Cambodia, and Viet Nam, national programmes should further scale up shorter rifamycin-based regimens including child-friendly formulations, and decentralize TPT delivery through primary care and community health workers [41–43]. Complementary strategies, such as digital adherence support and integration of TPT into HIV, maternal and child health, and primary care platforms, can further strengthen TPT implementation and completion [42].

Finally, TB-affected households in the Western Pacific Region often experience severe financial hardship, which can drive families deeper into poverty and limit their ability to meet basic needs such as food, housing, and education. National household cost surveys consistently identify income loss and direct non-medical expenses as major cost drivers [37, 44], with nutritional supplements representing a substantial share in some settings [36, 45, 46]. However, a recent regional baseline assessment found that social protection programmes across five high-burden countries in the Region remain fragmented and poorly accessible to people affected by TB, with coverage reaching only a small minority of eligible households [47]. In Lao PDR, the provision of nutritional counselling and support has been shown to mitigate catastrophic costs by reducing non-medical costs, while also contributing to early nutritional recovery, highlighting the importance of service integration at the point of care [48–50]. Similarly, a study from Cambodia demonstrated that ACF has the great potential to mitigate the costs incurred particularly before treatment [51]. These experiences underscore that mitigating catastrophic costs among TB‑affected households requires a multidimensional approach that addresses barriers to health‑care access while strengthening social, nutritional, and financial support mechanisms.

This study has several limitations. First, the analysis relied on country‑reported surveillance data which are subject to under‑reporting, misclassification, and variability in data completeness and quality across countries and over time. Second, several indicators, particularly those related to TB-HIV care, TPT and household costs, were available for only a subset of countries or years, limiting cross‑country comparability and trend interpretation. Third, as a descriptive analysis, this study did not assess causal relationships or formally evaluate the impact of specific interventions or policies. Finally, as TB case notifications are concentrated in a small number of high‑burden countries, including Indonesia, the Philippines and China, regional‑level indicators should be interpreted with caution. Despite these limitations, the study provides a comprehensive and up‑to‑date overview of TB epidemiology and programme performance in the Western Pacific Region, drawing on the best available data to inform regional priority actions.

## Conclusions

The Western Pacific Region stands at a pivotal moment in its efforts to end TB. Recent progress—including restoration of case notifications, improvements in diagnostic capacity, and expansion of preventive treatment—is encouraging; however, overall progress remains slow and uneven. Persistent gaps in case detection and notification, slower‑than‑needed scale‑up of preventive treatment, and suboptimal treatment outcomes among key vulnerable populations continue to constrain further gains. TB epidemiology in the Region remains highly heterogeneous, characterized by a high childhood TB burden in several Pacific settings, a predominance of older adults in low‑incidence economies, and a growing proportion of foreign‑born cases in countries receiving migrants. Accelerating progress toward TB elimination will require country‑tailored strategies that respond to diverse epidemiological contexts and risk‑group needs, alongside urgent action to intensify case detection, address under‑reporting, improve equitable access to quality care for vulnerable populations, and expand access to short‑course TPT. Above all, sustained investment in data‑driven, evidence‑based approaches adapted to national contexts will be critical to consolidate recent gains and achieve lasting impact on the TB epidemic in the Region.

## Data Availability

All data used in this study are publicly available from the World Health Organization Global Tuberculosis Programme and can be accessed through the WHO website: https://www.who.int/teams/global-programme-on-tuberculosis-and-lung-health/data

## References

[1] World Health Organization. Global Tuberculosis Report 2025. Geneva; 2025.

[2] Uplekar M, Weil D, Lonnroth K, Jaramillo E, Lienhardt C, Dias HM, et al. WHO’s new End TB Strategy. Lancet. 2015;385:1799–801. 10.1016/S0140-6736(15)60570-0. 25814376 10.1016/S0140-6736(15)60570-0

[3] World Health Organization Regional Office for the Western Pacific. Western Pacific Regional Framework to End TB: 2021–2030. Manila; 2021.

[4] Reid M, Agbassi YJP, Arinaminpathy N, Bercasio A, Bhargava A, Bhargava M, et al. Scientific advances and the end of tuberculosis: a report from the Lancet Commission on Tuberculosis. Lancet. 2023;402:1473–98. 10.1016/S0140-6736(23)01379-X. 37716363 10.1016/S0140-6736(23)01379-X

[5] World Health Organization. Consolidated guidance on tuberculosis data generation and use: module 1: tuberculosis surveillance. Geneva; 2024.

[6] Oh KH, Morishita F, Rahevar K, Yadav R-P, Tran HTG, Marks GB, et al. The Western Pacific Regional Framework to End TB: overview and critical reflection. IJTLD Open. 2025;2:64–72. 10.5588/ijtldopen.24.0608. 39959399 10.5588/ijtldopen.24.0608 PMC11827672

[7] World Health Organization. Global tuberculosis database. 2025.

[8] World Health Organization. Technical appendix: Methods used by WHO to estimate the global burden of TB disease for publication in the global tuberculosis report 2025. 2025.

[9] Morishita F, Viney K, Lowbridge C, Elsayed H, Oh KH, Rahevar K, et al. Epidemiology of tuberculosis in the Western Pacific Region: progress towards the 2020 milestones of the End TB Strategy. West Pac Surveill Response J. 2020;11:10–23. 10.5365/wpsar.2020.11.3.002. 10.5365/wpsar.2020.11.3.002 PMC8152824 34046237

[10] Oh KH, Yanagawa M, Morishita F, Glaziou P, Rahevar K, Yadav RP. Changing epidemic of tuberculosis amidst the COVID-19 pandemic in the Western Pacific Region: analysis of tuberculosis case notifications and treatment outcomes from 2015 to 2022. Lancet Reg Health West Pac. 2024. 10.1016/j.lanwpc.2024.101104. 38911260 10.1016/j.lanwpc.2024.101104 PMC11190483

[11] MacLean EL, Oh KH, Rahevar K, Nathanson CM, Korobitsyn A, Mitarai S, Kato S, Morishita F, Tran HTG, Yadav RPH; Tuberculosis diagnosis and laboratory services western pacific regional assessment consortium. Progress in tuberculosis diagnosis and laboratory services in the Western Pacific Region: a situational analysis of seven high tuberculosis burden countries. Trop Med Health. 2026. 10.1186/s41182-025-00898-z 42152159 10.1186/s41182-025-00898-z PMC13182110

[12] Oh KH, Quelapio MI, Morishita F, Rahevar K, Yanagawa M, Islam T. Progress on diagnosis and treatment of drug-resistant tuberculosis in line with World Health Organization recommendations in six priority countries in the Western Pacific Region. West Pac Surveill Response J. 2022;13:12–23. 10.5365/wpsar.2022.13.4.972. 10.5365/wpsar.2022.13.4.972 PMC9912284 36817504

[13] World Health Organization. Systematic screening for active tuberculosis: an operational guide. Geneva; 2015.

[14] World Health Organization. WHO consolidated guidelines on tuberculosis: module 2: screening: systematic screening for tuberculosis disease. Geneva; 2021. 33822560

[15] Eang MT, Satha P, Yadav RP, Morishita F, Nishikiori N, van-Maaren P, et al. Early detection of tuberculosis through community-based active case finding in Cambodia. BMC Public Health. 2012;12:469. 10.1186/1471-2458-12-469. 22720878 10.1186/1471-2458-12-469 PMC3489610

[16] Morishita F, Eang MT, Nishikiori N, Yadav R-P. Increased case notification through active case finding of tuberculosis among household and neighbourhood contacts in Cambodia. PLoS ONE. 2016;11:e0150405. 10.1371/journal.pone.0150405. 26930415 10.1371/journal.pone.0150405 PMC4773009

[17] Morishita F, Garfin AMCG, Lew W, Oh KH, Yadav R-P, Reston JC, et al. Bringing state-of-the-art diagnostics to vulnerable populations: the use of a mobile screening unit in active case finding for tuberculosis in Palawan, the Philippines. PLoS ONE. 2017;12:e0171310. 10.1371/journal.pone.0171310. 28152082 10.1371/journal.pone.0171310 PMC5289556

[18] Dakulala P, Kal M, Honjepari A, Morris L, Rehan R, Akena SP, et al. Evaluation of a population-wide, systematic screening initiative for tuberculosis on Daru island, Western Province, Papua New Guinea. BMC Public Health. 2024;24:959. 10.1186/s12889-024-17918-y. 38575948 10.1186/s12889-024-17918-y PMC10993525

[19] Innes AL, Martinez A, Hoang GL, Nguyen TBP, Vu VH, Luu THT, et al. Active case finding to detect symptomatic and subclinical pulmonary tuberculosis disease: implementation of computer-aided detection for chest radiography in Viet Nam. West Pac Surveill Response J WPSAR. 2024;15:1–12. 10.5365/wpsar.2024.15.4.1118. 39416596 10.5365/wpsar.2024.15.4.1118 PMC11473474

[20] Teo AKJ, Oh KH, Yanagawa M, Miller C, Falzon D, Kanchar A, et al. Programmatic approaches to screening for tuberculosis disease: a situational analysis of seven countries in the Western Pacific Region. Trop Med Health. 2025;53:185. 10.1186/s41182-025-00846-x. 41382194 10.1186/s41182-025-00846-x PMC12699892

[21] World Health Organization. WHO operational handbook on tuberculosis: module 2: screening: systematic screening for tuberculosis disease. Geneva; 2022. 33822560

[22] Emery JC, Dodd PJ, Banu S, Frascella B, Garden FL, Horton KC, et al. Estimating the contribution of subclinical tuberculosis disease to transmission: an individual patient data analysis from prevalence surveys. Elife. 2023;12:e82469. 10.7554/eLife.82469. 38109277 10.7554/eLife.82469 PMC10727500

[23] Nguyen T-A, Teo AKJ, Zhao Y, Quelapio M, Hill J, Morishita F, et al. Population-wide active case finding as a strategy to end TB. Lancet Reg Health - Western Pacific. 2024. 10.1016/j.lanwpc.2024.101047. 38827931 10.1016/j.lanwpc.2024.101047 PMC11143452

[24] Zhou D, Pender M, Jiang W, Mao W, Tang S. Under-reporting of TB cases and associated factors: a case study in China. BMC Public Health. 2019;19:1664. 10.1186/s12889-019-8009-1. 31829147 10.1186/s12889-019-8009-1 PMC6907198

[25] Islam T, van Weezenbeek C, Vianzon R, Garfin AMCG, Hiatt T, Lew WJ, et al. Market size and sales pattern of tuberculosis drugs in the Philippines. Public Health Action. 2013;3:337–41. 10.5588/pha.13.0094. 26393058 10.5588/pha.13.0094 PMC4463147

[26] World Health Organization Regional Office for the Western Pacific. Epidemiological Review for TB in the Philippines, 2022. Manila; 2022.

[27] World Health Organization. The second national TB inventory study in Indonesia. Global Tuberculosis Report 2024. 2024. https://www.who.int/teams/global-programme-on-tuberculosis-and-lung-health/tb-reports/global-tuberculosis-report-2024/featured-topics/the-second-national-tb-inventory-study-in-indonesia. Accessed 6 Sept 2025.

[28] World Health Organization. Consolidated guidance on tuberculosis data generation and use: module 1: tuberculosis surveillance: web annex C: record-linkage exercises. Geneva: World Health Organization; 2024.

[29] World Health Organization. WHO consolidated guidelines on tuberculosis: module 5: management of tuberculosis in children and adolescents. Geneva: World Health Organization; 2022. 35404556

[30] World Health Organization. WHO operational handbook on tuberculosis: module 5: management of tuberculosis in children and adolescents. Geneva: World Health Organization; 2022. 35404556

[31] Teo AKJ, Morishita F, Islam T, Viney K, Ong CWM, Kato S, et al. Tuberculosis in older adults: challenges and best practices in the Western Pacific Region. Lancet Reg Health Western Pacific. 2023. 10.1016/j.lanwpc.2023.100770. 37547037 10.1016/j.lanwpc.2023.100770 PMC10398605

[32] Teo AKJ, Rahevar K, Morishita F, Ang A, Yoshiyama T, Ohkado A, et al. Tuberculosis in older adults: case studies from four countries with rapidly ageing populations in the Western Pacific Region. BMC Public Health. 2023;23:370. 10.1186/s12889-023-15197-7. 36810018 10.1186/s12889-023-15197-7 PMC9942033

[33] Wong FSY, Morishita F, Oh KH, Tran HTG, Yadav R-P. Tuberculosis among foreign-born populations in the Western Pacific Region: emerging trends and analysis from 2008 to 2023. Trop Med Health. 2025;53:190. 10.1186/s41182-025-00866-7. 41419986 10.1186/s41182-025-00866-7 PMC12715926

[34] Engaging private health care providers in TB care and prevention: a landscape analysis (second edition). Geneva: World Health Organization; 2021.

[35] WHO consolidated guidelines on tuberculosis. Module 6: Tuberculosis and comorbidities. Geneva: World Health Organization; 2024. 38713785

[36] Chittamany P, Yamanaka T, Suthepmany S, Sorsavanh T, Siphanthong P, Sebert J, et al. First national tuberculosis patient cost survey in Lao People’s Democratic Republic: assessment of the financial burden faced by TB-affected households and the comparisons by drug-resistance and HIV status. PLoS ONE. 2020;15:e0241862. 10.1371/journal.pone.0241862. 33180777 10.1371/journal.pone.0241862 PMC7660466

[37] World Health Organization. National surveys of costs faced by tuberculosis patients and their households 2015-2021. Geneva: World Health Organization; 2023.

[38] Monthly Report of Tuberculosis Surveillance, Japan - December 2025. TB Surveillance Center, RIT, JATA.

[39] Seyedmehdi SM, Jamaati H, Varahram M, Tabarsi P, Marjani M, Moniri A, et al. Barriers and facilitators of tuberculosis treatment among immigrants: an integrative review. BMC Public Health. 2024;24:3514. 10.1186/s12889-024-21020-8. 39696110 10.1186/s12889-024-21020-8 PMC11658328

[40] World Health Organization. Guidance on social protection for people affected by tuberculosis. Geneva: World Health Organization; 2024.

[41] World Health Organization. WHO consolidated guidelines on tuberculosis Module 1: prevention - tuberculosis preventive treatment, second edition. Geneva; 2024. 39298638

[42] Oh KH, Teo AKJ, Yanagawa M, Kanchar A, Falzon D, Miller C, et al. Progress and challenges in tuberculosis preventive treatment in the Western Pacific Region: a situational analysis of seven high tuberculosis burden countries. Trop Med Health. 2025;53:122. 10.1186/s41182-025-00805-6. 40890887 10.1186/s41182-025-00805-6 PMC12403340

[43] Teo AKJ, Morishita F, Prem K, Eng S, An Y, Huot CY, et al. Where are the missing people affected by tuberculosis? A programme review of patient-pathway and cascade of care to optimise tuberculosis case-finding, treatment and prevention in Cambodia. BMJ Glob Health. 2023. 10.1136/bmjgh-2022-010994. 36921989 10.1136/bmjgh-2022-010994 PMC10030488

[44] Portnoy A, Yamanaka T, Nguhiu P, Nishikiori N, Baena IG, Floyd K, et al. Costs incurred by people receiving tuberculosis treatment in low-income and middle-income countries: a meta-regression analysis. Lancet Glob Health. 2023;11:e1640–7. 10.1016/S2214-109X(23)00369-8. 37734806 10.1016/S2214-109X(23)00369-8 PMC10522775

[45] Viney K, Itogo N, Yamanaka T, Jebeniani R, Hazarika A, Morishita F, et al. Economic evaluation of patient costs associated with tuberculosis diagnosis and care in Solomon Islands. BMC Public Health. 2021;21:1928. 10.1186/s12889-021-11938-8. 34688266 10.1186/s12889-021-11938-8 PMC8542301

[46] Youngkong S, Kamolwat P, Wongrot P, Thavorncharoensap M, Chaikledkaew U, Nateniyom S, et al. Catastrophic costs incurred by tuberculosis affected households from Thailand’s first national tuberculosis patient cost survey. Sci Rep. 2024;14:11205. 10.1038/s41598-024-56594-1. 38755216 10.1038/s41598-024-56594-1 PMC11099064

[47] Boccia D, Rahevar K, Carter DJ, Pescarini JM, Schwalb A, Islam T, et al. Towards universal social protection for people affected by tuberculosis in the Western Pacific Region: a social protection baseline assessment and policy entry points. Trop Med Health. 2026;54:47. 10.1186/s41182-025-00887-2. 41821111 10.1186/s41182-025-00887-2 PMC12980936

[48] Inthavong D, Elsayed H, Keonakhone P, Seevisay V, Souksanh S, Suthepmany S, et al. Does nutritional support contribute to mitigating the financial burden faced by TB-affected households? IJTLD OPEN. 2025;2:260–8. 10.5588/ijtldopen.25.0079. 40365024 10.5588/ijtldopen.25.0079 PMC12068456

[49] Inthavong D, Elsayed H, Keonakhone P, Seevisay V, Souksanh S, Suthepmany S, et al. The prevalence of undernutrition and associated risk factors in people with tuberculosis in Lao People’s Democratic Republic. PLoS ONE. 2025;20:e0324838. 10.1371/journal.pone.0324838. 40540510 10.1371/journal.pone.0324838 PMC12180643

[50] Inthavong D, Elsayed H, Keonakhone P, Seevisay V, Souksanh S, Suthepmany S, et al. Impact of nutritional counselling and support on body mass index recovery and treatment outcomes among tuberculosis patients in the Lao People’s Democratic Republic. Trop Med Infect Dis. 2025;10:198. 10.3390/tropicalmed10070198. 40711075 10.3390/tropicalmed10070198 PMC12300389

[51] Morishita F, Yadav R-P, Eang MT, Saint S, Nishikiori N. Mitigating financial burden of tuberculosis through active case finding targeting household and neighbourhood contacts in Cambodia. PLoS ONE. 2016;11:e0162796. 10.1371/journal.pone.0162796. 27611908 10.1371/journal.pone.0162796 PMC5017748

